# Use of a Robotic Device for the Rehabilitation of Severe Upper Limb Paresis in Subacute Stroke: Exploration of Patient/Robot Interactions and the Motor Recovery Process

**DOI:** 10.1155/2015/482389

**Published:** 2015-03-02

**Authors:** Christophe Duret, Ophélie Courtial, Anne-Gaëlle Grosmaire, Emilie Hutin

**Affiliations:** ^1^CRF Les Trois Soleils, Médecine Physique et de Réadaptation, Unité de Rééducation Neurologique, 77310 Boissise-le-Roi, France; ^2^Analyse et Restauration du Mouvement, Groupe Hospitalier Henri Mondor, Rééducation Neurolocomotrice, AP-HP, 94010 Créteil, France

## Abstract

This pioneering observational study explored the interaction between subacute stroke inpatients and a rehabilitation robot during upper limb training. 25 stroke survivors (age 55 ± 17 years; time since stroke, 52 ± 21 days) with severe upper limb paresis carried out 16 sessions of robot-assisted shoulder/elbow training (InMotion 2.0, IMT, Inc., MA, USA) combined with standard therapy. The values of 3 patient/robot interaction parameters (a guidance parameter: Stiffness, a velocity-related parameter: Slottime, and Robotic Power) were compared between sessions 1 (S1), 4 (S4), 8 (S8), 12 (S12), and 16 (S16). Pre/post Fugl-Meyer Assessment (FMA) scores were compared in 18 patients. Correlations between interaction parameters and clinical and kinematic outcome measures were evaluated. Slottime decreased at S8 (*P* = 0.003), while Guidance decreased at S12 (*P* = 0.008). Robotic Power tended to decrease until S16. FMA scores improved from S1 to S16 (+49%, *P* = 0.002). Changes in FMA score were correlated with the Stiffness parameter (*R* = 0.4, *P* = 0.003). Slottime was correlated with movement velocity. This novel approach demonstrated that a robotic device is a useful and reliable tool for the quantification of interaction parameters. Moreover, changes in these parameters were correlated with clinical and kinematic changes. These results suggested that robot-based recordings can provide new insights into the motor recovery process.

## 1. Introduction

After stroke, most patients have residual upper limb (UL) motor impairments, leading to long-term limitations in function which impact quality of life [1, 2]. Motor recovery is often poor with only one-third of all stroke patients regaining some dexterity within six months [3].

Over the last 2 decades, many studies have investigated the neuroplastic changes which occur after the acute event as well as optimal strategies to restore lost motor function. This growing body of evidence has demonstrated that large numbers of movement repetitions [4–6], carried out within intense [7–9] and specific task-oriented [10, 11] training programs, are required to drive optimal neuroplastic changes and to improve function. This scientific knowledge has stimulated the development and the use of technological devices, referred to as rehabilitation robots, to address the need for intensive training. This training is carried out under the supervision of therapists. Advanced robotic systems can provide repetitive, reproducible, and interactive forms of physical therapy which can be quantified. Since the first clinical studies of the MIT-MANUS robot [12] at the Massachusetts Institute of Technology (MIT), this innovative therapeutic tool has been clinically studied for the rehabilitation of the paretic upper limb, mainly after stroke. There are a multitude of studies of patients in the acute/subacute phase of stroke recovery [12–16] as well as in the chronic phase [17–20]. The results were very promising, showing that robotic therapy is safe and well tolerated [12, 15, 19, 20] and that it has a positive impact, improving motor impairments. These results led to the endorsement of the use of upper extremity robotics in the 2010 guidelines of the American Heart Association for Stroke Care [21].

Robot-mediated training is highly repetitive in nature. Indeed, such systems allow stroke patients, including those with severe impairment, to repeat movements hundreds of times. This is physically impossible using usual treatment methods [22]. This feature is mostly due, in the most advanced systems, to the use of active robotic controllers [23]. The principle paradigm implemented to date consists of performance-based algorithms that enable the robot to adjust the mechanical assistance provided during the training session according to the patient's motor performance. Most robotic devices use assisted-as-needed programs, the aim of which is to provide only as much assistance as the patient requires to complete the task. However, a potential problem with most robotic controllers is the provision of excessive assistance. This can encourage patients to minimize their efforts, resulting in a reduction of experience-dependent plasticity [24, 25]. There is currently a lack of literature regarding how humans and particularly stroke survivors interact with robotic-therapy devices.

In addition to therapeutic effects, some robots can assess motor performance during robot-assisted tasks by recording biomechanical parameters (mainly position and speed of the hand). Some robots can provide new insights into the effectiveness of treatment through the capture of motion kinematics [26–28] and some can measure and record patient-robot interactions during training. Thus, some devices can continuously track motor performance through the measurement of specific indicators, including the patient's actual level of participation. Such data are difficult to obtain in usual care.

The aim of the present study was to investigate patient-robot interactions and to analyze changes and potential correlations with clinical and kinematic outcome measures during an upper limb robot-assisted training program in subacute stroke patients with severe motor impairments.

## 2. Materials and Methods

### 2.1. Participants

From October 2010 to March 2013, 48 inpatients involved in the upper limb robotic program were screened for inclusion in this observational study. These patients had undergone an upper limb robot-assisted rehabilitation program administered as part of usual care for moderately to severely motor impaired inpatients admitted to the Neurorehabilitation Unit at Les Trois Soleils Rehabilitation Center (Boissise-le-Roi, France). 25 stroke survivors (13 females, age 55 ± 17 (19–88) years, 21 ischemic strokes, and 4 hemorrhagic strokes) were enrolled. The inclusion criteria were the following: being over 18 years old, with moderate to severe upper limb paresis defined by a low motor score (≤35 on the Fugl-Meyer Assessment (FMA) scale [29, 30]), being in the subacute phase of stroke (time since stroke, 52 ± 21 days), with a single lesion confirmed on CT scan or MRI, and with sufficient understanding to participate in rehabilitation exercises (see Table 1). Finally, patients had to have carried out the whole upper limb robot-mediated training using the assistive robotic mode (patients who had used the passive and active modes within some robotic sessions were not included). This observational study was approved by the “CPP Ile de France 1” Ethics Committee.

### 2.2. Interventions and Apparatus

#### 2.2.1. Apparatus

**Figure 1:**
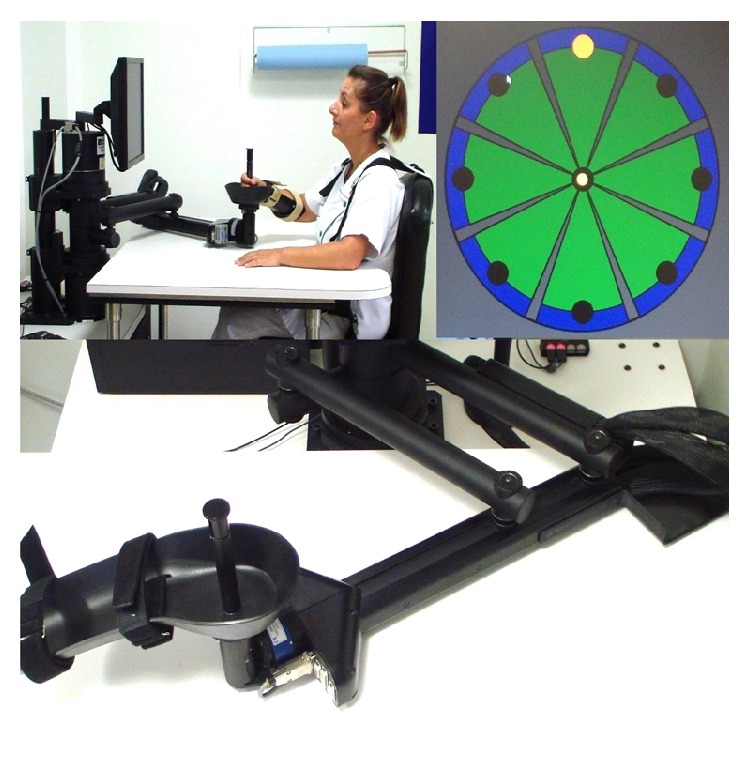
Experimental setup (upper left, setup with an individual; upper right, “clock exercise”; lower, Arm robot).

The InMotion 2.0 Arm robot (Interactive Motion Technologies, Inc., Watertown, MA, Figure 1), the commercial version of the MIT Manus, was used for the study [31]. This device is a 2 translational degrees-of-freedom planar robot that emphasizes shoulder (flexion/extension) and elbow (flexion/extension) movements in the horizontal plane. It was designed to have low intrinsic endpoint impedance with a low inertia. This device has several treatment modes, including an adaptive (or assist-as-needed) program using a performance-based algorithm that adjusts forces to assist or challenge the patient's movement according to his/her motor performance. Particularly, if a task cannot be completed volitionally, the robot provides assistance to reach the target.

The point-to-point unconstrained reaching program is mainly used for the evaluation of motion kinematics.

#### 2.2.2. Interventions

All the patients underwent 16 sessions of upper limb robot-assisted training in addition to their usual stroke rehabilitation program. Each session lasted for 45 minutes, 4 days per week.

During the training, the patient was seated on an adjustable chair in front of a monitor which displayed goal-directed exercises. The trunk was restrained by a harness to decrease compensatory movements. The paretic limb was supported at the elbow by a splint. The shoulder was in 45° elevation and the elbow slightly flexed. The wrist was in a neutral position and the fingers were placed around the handle (Figure 1).

**Figure 2:**
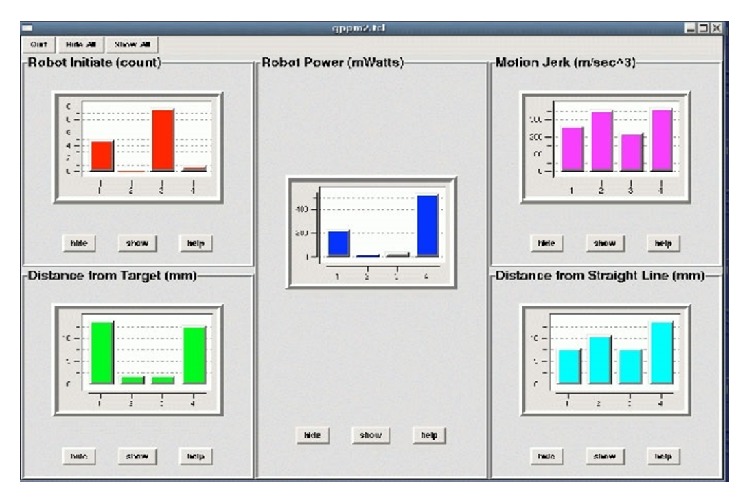
Performance feedback (displayed after each block of 80 movements).

The patient held the robot handle to perform the exercises. The motor tasks involved point-to-point gravity-compensated reaching towards 8 visual targets displayed in the 8 compass directions on the monitor and presented in a clockwise order. Each target was 14 cm from the center of the monitor. The patient was instructed to perform as many accurate movements as possible in the allocated training time. The training consisted of series of 320 repetitions (4 blocks of 80 movements). Patients were allowed a 1-to-3-minute break after each block. A summary graph also displayed patient's performance and several interaction parameters after each block (Figure 2).

During the training, patients performed an average of 614 ± 250 movements during the first session (S1), 780 ± 271 movements at the midpoint of the training (session 8, S8), and 857 ± 342 movements during the last session (S16) (see Table 2).

Standard care for the paretic upper limb consisted of one-hour occupational therapy sessions 5 days per week; this program involved passive stretching within submaximal ranges of motion with inhibition of spasticity if necessary [32], active assisted movements, reaching movements with or without elbow support, and grasping tasks that were tailored to the abilities of each patient [33].

This comprehensive program of care also included one-hour daily (5 days a week) sessions of physical therapy based on lower limb rehabilitation (without upper limb therapy) and, if necessary, one hour of speech therapy 3-4 times a week.

### 2.3. Robot-Based Outcome Measures

The robotic device measures several parameters related to patient-robot interactions, indicating the level of assistance and/or challenge provided by the robot while the patient performed the reaching task. The values recorded after the 80th movement (out of 320) were analyzed for the 25 patients. This was because, during the first 80 movements, these parameters are frequently adjusted by the robot (every 16 movements).

The following parameters were analyzed.

*Stiffness* is a parameter of lateral guidance. The robot adapts the stiffness of the side walls, thus regulating the amount of guidance given to the patient to produce straight movements. As patients get better at aiming, the amount of side guidance is reduced to challenge the patient to make even straighter movements. Stiffness is defined as force/displacement and is measured in Newton/meters. The default stiffness of 200 N/m was used to begin with and the adaptive algorithm then adjusted the stiffness according to the movements performed by the patient.

*Slottime* is the time allotted to the patient to achieve the task. The initial time allowed is 2 seconds. As the patient moves faster, the time is gradually decreased to 1 second. This is a velocity-related parameter.

*Robot Power* is defined as force ∗ velocity, calculated from the force transducer measurements (force) and the position measurements in the direction of the target (velocity). If the patient performs the whole movement without assistance, the value will be close to zero, that is, minimum interaction force registered in the transducer.

The interaction parameters were analyzed at S1, S4, S8, S12, and S16.

In addition to the interaction parameters, 2 kinematic metrics calculated from trajectory recordings carried out during a robot-based evaluation (80 movements toward 8 targets) were analyzed at S1 and S16 in 19 patients (data missing for 6 patients); the mean velocity (m/s) and the movement accuracy were calculated as the mean deviation from the straight line (m).

### 2.4. Clinical Outcomes

Motor impairment was measured before the first session and after the last session using the upper extremity motor section of the Fugl-Meyer Assessment (FMA) in 18 patients (7 patients with incomplete data). The FMA scale measures the ability to move the paretic arm, including items related to movements of the shoulder, elbow, wrist, and hand. Each item is rated on a 3-point scale (maximum score, 66 points).

### 2.5. Statistical Analysis

The values of the 3 interaction parameters were compared across sessions (S1, S4, S8, S12, and S16) in 25 patients (Table 2; ANOVA, post hoc Tukey test). A descriptive analysis was performed to compare FMA scores between S1 and S16 in 18 patients (*t*-test).

Pearson's coefficients were used to explore correlations between the interaction parameters, changes in FMA score, and selected kinematic parameters, as well as potential correlations between the 3 interaction parameters (S1 versus S16).

## 3. Results

**Figure 3:**
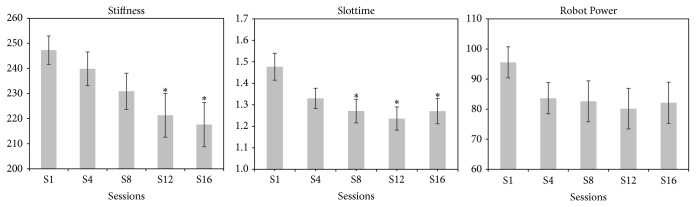
Changes in interaction parameters over the training period. ^*^: versus S1, *P* < 0.05.

Slottime decreased at S8 (*P* = 0.003). Stiffness decreased at S12 (*P* = 0.008). There was a trend towards a decrease in Robot Power (Figure 3).

The FMA score improved significantly from S1 to S16 (+49%, *P* = 0.002).

**Figure 4:**
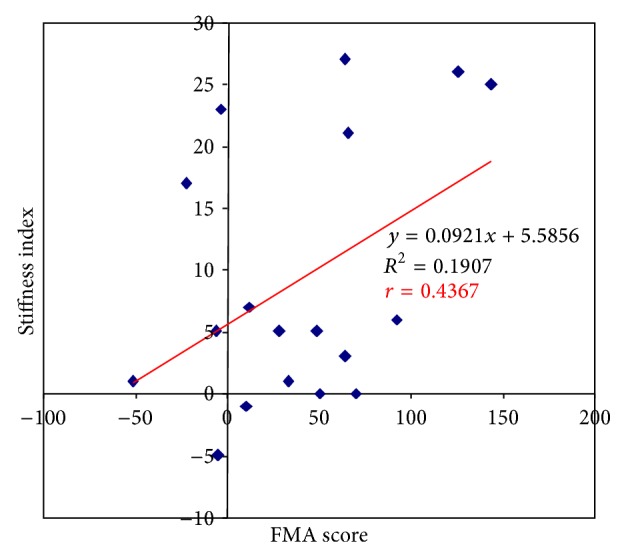
Correlation between FMA score and changes in Stiffness.

**Figure 5:**
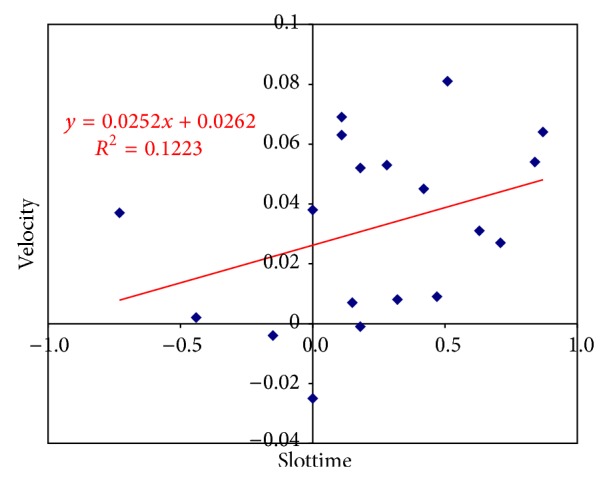
Correlation between Slottime and velocity.

The regression analysis showed that the change in FMA score was correlated with the change in Stiffness (*r* = 0.4, *P* = 0.003) (Figure 4) but not with the other interaction parameters. There was a good correlation (*r* = 0.35) between Slottime and the mean change in velocity. There was no correlation between Stiffness and changes in movement accuracy. Finally, there was a negative correlation between the change in Stiffness and the change in Slottime (*r* = −0.6, *P* = 0.001) (Figure 5).

## 4. Discussion

The present study is, to our knowledge, the first to analyze how patients with moderate to severe motor impairment following stroke interact with a rehabilitation robot during upper limb training carried out as part of a stroke rehabilitation program in the subacute phase. Moreover, the nature of the interactions and how they changed during the robot-mediated program were evaluated. Potential correlations with clinical scores and kinematic metrics were also analyzed.

The evaluation of interaction parameters, which is difficult or impossible in usual care, appears to be of critical interest because it affords an insight into the level of active participation of patients, as well as both quantitative and qualitative motor performance. In fact, some recent results from studies of robotic devices for gait training [34–36] demonstrated that the patient's level of engagement is a crucial determinant of robot-mediated rehabilitation. Such rehabilitation is more effective when the user actively participates in the movement, and the use of full and passive guidance could have a negative impact on recovery in stroke patients. A previous study showed that continuous passive motion using an upper limb robotic system did not provide any advantage over conventional therapy [37].

The present study confirmed that the robot used for the rehabilitation program was a truly interactive device, since it adjusted its action according to changes which occurred in the patient's motor performance during the training. This approach was pioneering but it fully depended on the design of the robot used in the study which enabled the recording of and easy access to several interaction parameters; indeed, we were able to specify the type of assistance provided to the patient by the robot, showing that interactions were multimodal. The robot recorded kinematic data (mainly velocity and position) and, through the use of a performance-based algorithm, applied forces laterally, which guided the hand to assist aiming (Stiffness), as well as “longitudinally” to facilitate reaching to the target (Robotic Power). An additional and intricate parameter took into account the time taken to reach the target in order to challenge the patient to perform the task even faster (Slottime). These parameters were used to create both assistive and challenging effects. The results demonstrated that patient/robot interactions changed “positively” over the training period, following dynamic and differentiated processes. In fact, the velocity-related parameter (Slottime) decreased earlier than the lateral guidance parameter (Stiffness). The decrease in Robotic Power was smaller. This latter finding could be explained by the characteristics of the patients and the duration of the training. Indeed, the patients all had moderate to severe motor impairments and it was possible that the 5 weeks of training were insufficient for significant reductions in this type of robotic assistance to occur.

This study also showed that changes in the interaction parameters were well correlated with clinically evaluated motor performance and certain kinematic parameters (velocity). These results might indicate that these parameters could be reliable indicators of objective motor performance. This is novel because no other study has used such a correlation approach to evaluate interaction.

Another finding is that, above providing movement support and modeling the time course of changes in motor performance, the robot challenged the patient, thus promoting further motor improvement (by decreasing lateral guidance and the time allocated to perform the task). This result is promising as it relates to the need to design robotic algorithms which take into account the fact that the human motor control system might reduce its participation when the controller is too compliant [25]. The present results suggest that robotic rehabilitation based on an adaptive program optimized the participation of patients, including those with severe motor impairment, thus resulting in a potential enhancement of experience-dependent plasticity.

As the patients included in this study were in the subacute phase of stroke, these results might provide an insight into the process of motor recovery in the early stages after stroke. In fact, the results suggest that movement velocity recovered before accuracy. This is concordant with previous work [38, 39]. The negative correlation between lateral guidance and the velocity-related parameter also suggested that there may be a speed/accuracy tradeoff in motor performance.

The study also highlighted that robotic devices could provide a compliment to clinical scales by quantifying motor performance, including longitudinal measurements of active participation. This latter parameter is useful to gain an understanding of the recovery process, besides motion kinematics which are typically evaluated in robotic studies [40, 41]. Even if the discriminant validity of kinematic variables as measures of UL impairment is still unclear, it appears that kinematic assessments extend clinical scales, assessing sensorimotor function in a more objective and reliable way and in repeatable conditions [42, 43].

The results reported should be interpreted with caution because the sample was small and the study was observational. Other limitations include the fact that data were incomplete for some patients.

## 5. Conclusions

This study used a novel approach to evaluate motor performance in the subacute phase of stroke. The results demonstrated that patient/robot interaction parameters are valid, relevant, and reliable variables for the quantification of active participation and motor performance in subacute stroke patients. Indeed, these parameters were correlated with clinical scores and with some kinematic parameters. These findings suggested that the analysis of patient/robot interactions might provide new insights into the motor recovery process.

## Figures and Tables

**Table 1:** Patient demographics.

Characteristics (*n* = 25)	
Gender (male/female)	12/13
Mean age ± SD (years)	55.5 ± 17
Time since stroke, mean ± SD (days)	52.2 ± 21.6
Type of stroke (H/I)	4/21
FMA score S1 (*n* = 18) mean ± SD [range]	19 ± 8.5 [7–35]
FMA score S16 (*n* = 18) mean ± SD [range]	28 ± 15.3 [9–57]

H, hemorrhagic; I, ischemic; FMA, Fugl-Meyer Assessment; SD = standard deviation.

**Table 2:** Changes in interaction parameters and number of movements from S1 to S16.

*n* = 25	S1	S4	S8	S12	S16
Stiffness (N/m) mean ± SD	247 ± 28	240 ± 34	231 ± 16	221 ± 44^a^	218 ± 44^a^
Slottime (s) mean ± SD	1.48 ± 0.31	1.33 ± 0.23	1.27 ± 0.28^a^	1.24 ± 0.27^a^	1.27 ± 0.29^a^
Robot (active) Power (mwatt) mean ± SD	95.5 ± 26	83.6 ± 26	82.6 ± 34	80.2 ± 34	82 ± 34
Number of movements mean ± SD	614 ± 250		780 ± 271		857 ± 342

^a^Versus S1, *P* < 0.05.
